# Three‐Phase Photocatalysis for the Enhanced Selectivity and Activity of CO_2_ Reduction on a Hydrophobic Surface

**DOI:** 10.1002/anie.201908058

**Published:** 2019-09-04

**Authors:** Ang Li, Qian Cao, Guangye Zhou, Bernhard V. K. J. Schmidt, Wenjin Zhu, Xintong Yuan, Hailing Huo, Jinlong Gong, Markus Antonietti

**Affiliations:** ^1^ Department of Applied Physics Nanjing University of Science and Technology Xiaolingwei street 200 Nanjing Jiangsu 210094 China; ^2^ Max Planck Institute of Colloids and Interfaces 14476 Potsdam Germany; ^3^ Key Laboratory for Green Chemical Technology of Ministry of Education School of Chemical Engineering and Technology Tianjin University Collaborative Innovation Center of Chemical Science and Engineering (Tianjin) Weijin Road 92 Tianjin 300072 China; ^4^ School of Chemical and Environmental Engineering Shanxi Datong University Xingyun street 405 Datong Shanxi 037009 China

**Keywords:** carbon nitride, CO_2_ reduction, hydrophobic material, photocatalysis, three-phase catalysis

## Abstract

The photocatalytic CO_2_ reduction reaction (CRR) represents a promising route for the clean utilization of stranded renewable resources, but poor selectivity resulting from the competing hydrogen evolution reaction (HER) in aqueous solution limits its practical applicability. In the present contribution a photocatalyst with hydrophobic surfaces was fabricated. It facilitates an efficient three‐phase contact of CO_2_ (gas), H_2_O (liquid), and catalyst (solid). Thus, concentrated CO_2_ molecules in the gas phase contact the catalyst surface directly, and can overcome the mass‐transfer limitations of CO_2_, inhibit the HER because of lowering proton contacts, and overall enhance the CRR. Even when loaded with platinum nanoparticles, one of the most efficient HER promotion cocatalysts, the three‐phase photocatalyst maintains a selectivity of 87.9 %. Overall, three‐phase photocatalysis provides a general and reliable method to enhance the competitiveness of the CRR.

## Graphical abstract

**A photocatalytic CO_2_ reduction reaction** (CRR) in aqueous solution is a promising reaction route, while severe H_2_ generation (HER) greatly limits its selectivity and activity. A strategy to obtain a three‐phase contact of CO_2_ (gas), H_2_O (liquid) and catalyst (solid) is described to overcome the mass transfer limitation of CO_2_, elevate the CO_2_ concentration on the catalyst surface, restrain the HER, and enhance the activity and selectivity of the CRR.

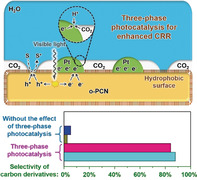

The ever‐increasing worldwide consumption of fossil fuels depletes these finite natural resources, but more urgently leads to overproduction of the greenhouse gas carbon dioxide (CO_2_).^[1]^ Hence, solutions to reduce CO_2_ emissions or, at best, utilization of CO_2_ in a sustainable energy cycle are highly relevant. As such, a photocatalytic CO_2_ reduction reaction (CRR) is in principle an efficient way to convert CO_2_ into valuable carbon derivatives (e.g. CO, CH_4_, C_2_H_4_, etc.) for energy supply.^[2]^ Ideally, the hydrogen source for the CRR should be supplied by water. In aqueous solutions the competing hydrogen evolution reaction (HER), that is, the direct water reduction, is dominant, resulting in most cases in low selectivity and activity of the CRR.^[3]^ Moreover, metal nanoparticles with a large work function (e.g. platinum, palladium, etc.^[4]^) are usually loaded onto photocatalysts to collect electrons for the acceleration of reduction reaction. However, most of the metals are more likely to promote the HER, causing a further decrease of the CRR pathway.^[5]^

Substantial efforts have been invested to suppress the HER and enhance CRR performance, including development of new materials,^[6]^ adjusting defect density,^[7]^ tailoring morphology^[8]^ and grain‐boundaries,^[9]^ etc. For instance, the materials related to complex/semiconductor hybrid photocatalysts achieved exciting activity and selectivity for CO_2_ reduction recently.^[10]^ As reported in 2017, Kuriki et al. developed the carbon nitride nanosheet, modified by supramolecular complex and Ag nanoparticles, elevating the CRR selectivity to a very high value of up to 98 %.^[11]^

Gradually, researchers realized the significance of catalyst accessibility for high concentrations of CO_2_ molecules, which is even more important in aqueous solution. Numerically, the ratio of CO_2_ to H_2_O molecules is 1:1300 at 1 atm pressure.^[3a]^ While protons (H^+^) are readily available in aqueous solutions by water ionization, the supply of CO_2_ molecules to the catalyst surface is limited by their low concentration and slow diffusivity. Raciti et al. confirmed that the concentration of CO_2_ molecules on the catalyst surface can even be completely depleted under strong reaction driving force.^[12]^ Such a limitation constitutes a significant hurdle for the CRR. Thus, it is urgent to reduce the H^+^ concentration and increase CO_2_ concentration on the catalyst surface to overcome the transfer limitations.

Herein, we overcome the mass‐transfer limitation of CO_2_ by creating a photocatalyst with a hydrophobic surface, which facilitates an efficient three‐phase contact of CO_2_ (gas), H_2_O (liquid), and catalyst (solid).^[3a]^ Like that, highly concentrated gas‐phase CO_2_ molecules can be delivered to the catalyst surface directly (Figure 1). With higher CO_2_ and lower H^+^ surface concentration, the HER can be obviously suppressed, while the performance of the CRR is improved. Even when loaded with platinum (Pt), one of the most efficient electron collection and HER promotion cocatalysts, the hydrophobic surface obviously restrains the HER and increases the availability of electrons for the CRR.

**Figure 1:**
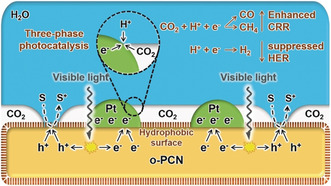
Scheme of the three‐phase photocatalyst Pt/*o*‐PCN and the mechanism of three‐phase photocatalysis. S represents the hole sacrificial agent (Na_2_S and Na_2_SO_3_ here),^[13]^ and S^+^ represents the sacrificial agent which is oxidized by holes.

The three‐phase photocatalyst presented here is a hydrophobic polymeric carbon nitride nanosheet loaded with Pt particles (Pt/*o*‐PCN). Carbon nitride is a stable material with suitable band structure for visible‐light absorption.^[14]^ The first reliable CO_2_ reduction catalyzed by carbon nitride was reported in 2013,^[15]^ and since then carbon nitride has been widely researched as a wonderful visible‐light response material for the CRR.^[10]^ The polymeric carbon nitride (PCN) is a two‐dimensional thin carbon nitride nanosheet (Figure 2 a), which can provide a large surface area to enrich active sites and shorten the diffusion distance of charges.^[16]^ Specifically, the surface area of a PCN‐based catalyst is approximately 16 times higher than bulk carbon nitride (see Figure S1 in the Supporting Information). In a following step, the surface of PCN is modified by polymer grafting,[^3a^, ^17^] and is stable under visible irradiation (see section 9 of methods in the Supporting Information and Figure S7), and the resulting hydrophobic PCN is denoted as *o*‐PCN (Figure 2 b). Pt nanoparticles are finally loaded onto the surface of *o*‐PCN to form Pt/*o*‐PCN (Figure 2 d) using the method of in situ photo‐loading.^[2]^ This approach facilitates a tight connection of carbon nitride and Pt exactly at the point of optimal electron transfer, and thus decreases the complex interface resistance between them. Pt functions as a reduction cocatalyst to collect electrons generated from carbon nitride, and delivers them to reactants. The enriched electrons on Pt may significantly strengthen the driving force of the targeted multielectron reduction half‐reaction.^[18]^

**Figure 2:**
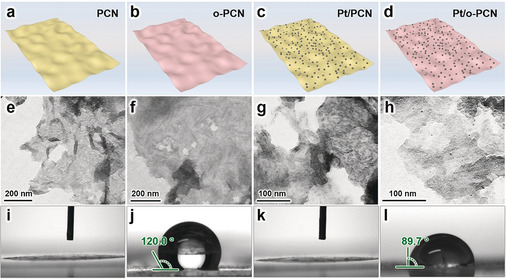
a–d) Models of all the photocatalysts including a) PCN, b) *o*‐PCN, c) Pt/PCN and d) Pt/*o*‐PCN. e–h) TEM images of e) PCN, f) *o*‐PCN, g) Pt/PCN, and h) Pt/*o*‐PCN. i–l) Contact angle measurement of water on i) PCN, j) *o*‐PCN, k) Pt/PCN and l) Pt/*o*‐PCN.

More specifically, the Pt/*o*‐PCN was synthesized in three steps. At first, PCN was synthesized by a calcination method with cyanuric acid and melamine as precursors at 550 °C.^[19]^ Secondly, PCN was modified with a polymer to create a hydrophobic surface.[^3a^, ^17^, ^20^] PCN was functionalized with ene‐modified poly(glycidyl methacrylate) (PGMA) (ene‐PGMA) under visible light irradiation. PCN produces radicals by light excitation, and results in an addition reaction between the PCN surface and the double‐bond on ene‐PGMA. Moreover, it is worth mentioning that the ene‐PGMA/PCN system remained stable under visible‐light irradiation for 48 hours. Subsequently, hydrophobic 1H, 1H, 2H, 2H‐perfluorodecanethiol (pFDe) was introduced to ene‐PGMA/PCN by a thiol‐epoxy reaction^[20b]^ between the thiol group in pFDe and the epoxides in the PGMA sidechains, leading to *o*‐PCN with a hydrophobic surface. Finally, Pt was loaded onto *o*‐PCN by the in situ photo‐loading method.^[2]^ To efficiently and randomly load Pt nanoparticles onto *o*‐PCN, acetone, which can well disperse *o*‐PCN, was used as the solvent during the photo‐loading. Ethanol and platinum bis(acetylacetonate) (Pt(acac)_2_) were dissolved in acetone, followed by irradiation with visible light under the pressure of 0.13 bar for 18 hours. Under irradiation, electrons and holes were generated from *o*‐PCN and migrated to the surface. Holes were consumed by ethanol, while electrons were delivered to Pt(acac)_2_ to form Pt particles (see Figure S2 a). Thus, only Pt(acac)_2_ complexes in contact with the exit sites of the *o*‐PCN surface were reduced to form Pt particles (see Figure S2 a). In this way Pt particles were formed in situ on the surface of *o*‐PCN, resulting in a tight connection between Pt and *o*‐PCN (see Figure S2 b).

For referencing, a benchmark catalyst was synthesized by directly loading Pt particles onto the commonly used unmodified PCN with a hydrophilic surface (Pt/PCN; Figure 2 c). Water, ethanol, and chloroplatinic acid (H_2_PtCl_6_) were used as solvent, holes sacrificial agent, and Pt source, respectively, and the mixture was also irradiated by visible light under 0.13 bar for 18 hours for the in situ loading of Pt particles onto the surface of PCN (see Figure S2 c,d).

The transmission electron microscope (TEM) images are shown in Figures 2 e–h. The image of PCN (Figure 2 e) confirms the successful synthesis of a two‐dimensional nanostructure. The *o*‐PCN shows a similar two‐dimensional morphology with a smoother surface (Figure 2 f), which may be attributed to the modification with polymer. From the TEM images of Pt/PCN (Figure 2 g; see Figure S3 a) and Pt/*o*‐PCN (Figure 2 h; see Figure S3 c), it can be observed that Pt particles are uniformly distributed on both PCN and *o*‐PCN to form well‐constructed Pt/PCN and Pt/*o*‐PCN, respectively. The results of inductively coupled plasma mass spectrometry (ICP‐OES) suggested Pt loadings of 2.32 and 2.61 wt % on Pt/PCN and Pt/*o*‐PCN, respectively, and they are similar enough to avoid the influence of mass loading on the catalytic activity.^[16b]^ Additionally, statistics based on TEM images suggest that the average sizes of Pt particles on Pt/PCN and Pt/*o*‐PCN are similar to each other (ca. 2.2 nm; see Figure S3), thus eliminating the influence of the particle size on the catalytic activity.^[16b]^

The surface properties relating to water wetting were investigated by contact‐angle measurement (Figures 2 i–l). Figures 2 i and k suggest that both PCN and Pt/PCN show significant hydrophilicity as water is completely spread out on them. Figure 2 j shows a contact angle of 120.0°, illustrating the hydrophobic surface of *o*‐PCN. After loading of Pt to form Pt/*o*‐PCN, the surface remains hydrophobic, with only a slightly weakened hydrophobicity resulting from the protruding Pt‐spots (Figure 2 i). The diminution of hydrophobicity is highly relevant, as it can be attributed to the hydrophilicity of Pt particles which are contacted with water, as shown in Figure 1.

Other characterizations were performed to further prove and investigate the chemical composition, structures, and photocatalytic properties (Figure 3). The X‐ray diffraction (XRD) pattern of PCN (Figure 3 a) shows the typical peaks which can be attributed to the (100) and (002) planes of carbon nitride. *o*‐PCN, Pt/PCN, and Pt/*o*‐PCN show similar XRD patterns to that of PCN (Figure 3 a), suggesting that the surface modification and Pt loading do not affect the crystallinity of carbon nitride. The peaks of Pt cannot be recognized from the XRD patterns of Pt/PCN and Pt/*o*‐PCN, owing to the low loading and small particle size of Pt.^[21]^ Thus, the presence of Pt was further evidenced by X‐ray photoelectron spectroscopy (XPS). The results of Pt 4f XPS analysis (Figure 3 b) show two peaks at about 75.4 and 72.3 eV, which can be attributed to Pt 4f_5/2_ and Pt 4f_7/2_ electrons,^[22]^ respectively, conforming the successful loading with Pt particles. Compared with the Pt 4f_5/2_ and Pt 4f_7/2_ binding energies of pure Pt (usually 73.8 and 70.4 eV, respectively^[22b]^), an appreciable shift to higher values was observed in Pt/PCN and Pt/*o*‐PCN, suggesting a strong interaction between the Pt particles and carbon nitride. The similar binding energy of Pt electrons in Pt/PCN and Pt/*o*‐PCN suggests that Pt particles loaded on both hydrophilic and hydrophobic surface share a similar electronic environment.^[23]^ The structures of carbon nitride substrates were analyzed by XPS spectra of N 1s, and can be divided into four peaks appearing at binding energies of about 398.3, 399.5, 400.6, and 404.4 eV (Figure 3 c).^[24]^ The dominant peaks at 398.3 and 399.5 eV are derived from the sp^2^‐hybridized aromatic N bonded to C atoms in the triazine units (C−N=C) and the tertiary N bonded to C atoms (N−(C)_3_), respectively (see Figure S4).^[24]^ The peak at 400.7 eV originates from the amino groups (C−N−H),^[24d]^ resulting from structural defects and incomplete condensation during the polymerization process (see Figure S4).[^14^, ^24a^] The contribution at about 404.2 eV originated from the positive over‐charging effect in the heterocycles owing to the protonation of carbon nitride.^[25]^ Figure 3 c shows that the N binding energies of PCN, *o*‐PCN, Pt/PCN, and Pt/*o*‐PCN are very close to each other, and the experimental ratios of nitrogen species in all catalysts are similar as well, indicating the unchanged structure of carbon nitride substrate during polymer modification and Pt loading process. Subsequently, the ultraviolet/visible (UV/Vis) optical absorption spectra (Figure 3 d) show that all the catalysts can absorb not only UV but also blue visible light, thus indicating a more sufficiently utilization of solar energy.^[26]^ The polymer modified surface may influence the light absorption within the whole UV/Vis range. Here we use the absorbance as the parameter to characterize the light absorption, since the absorbance can clearly reflect the difference of light absorption within the whole range.^[27]^ Compared with PCN and *o*‐PCN, both Pt/PCN and Pt/*o*‐PCN show enhanced light absorption in the visible region, but this enhancement resulting from the continuous energy bands of Pt does not contribute to the photocharge‐generating absorption.^[18a]^ The main functions of Pt are collection of electrons and promotion of reduction half‐reaction.^[28]^ Additionally, the efficiency of charge separation can be investigated by photoluminescence (PL) spectra (Figure 3 e), with stronger PL intensity indicating poorer stability of the separated charges.[^1b^, ^2^, ^28^] Figure 3 e shows that the intensity of PCN and *o*‐PCN is similar, suggesting that the polymer modification has no obvious effect on the charge separation. The significantly weaker PL intensity of Pt/PCN and Pt/*o*‐PCN indicates that Pt loading can significantly improve the efficiency of charge separation, which provides evidence that Pt is capable of electron collection.^[24c]^ As a summary of the analytics, Pt/PCN and Pt/*o*‐PCN share a similar local structure, electronic state of Pt, structure of carbon nitride substrate, size of Pt particles, properties of light absorption, and charge separation (Figure 3), while the only obvious difference lies in the presence of fluorophilic and CO_2_‐dissolving patches as reflected by the highly reduced wettability with water (Figure 2 k,l).^[29]^

**Figure 3:**
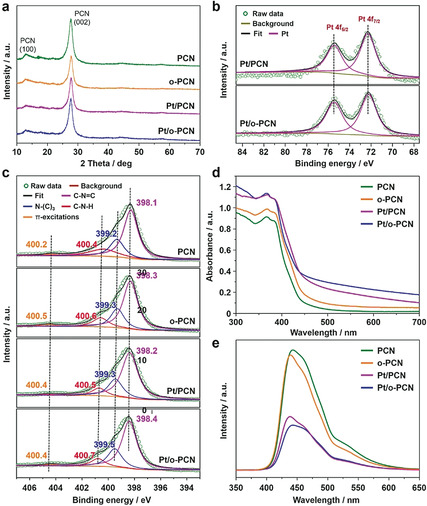
Analytical data of all the samples: a) XRD patterns. b) Pt 4f XPS analysis. c) N 1s XPS analysis. d) UV/Vis optical absorption spectra. e) PL spectra (wavelength of excitation light is 350 nm).

The photoactivity and selectivity of all catalysts were investigated in aqueous solution under visible‐light irradiation (for details see section 6 of methods). H_2_ is generated through the HER, while carbon derivatives are generated through the CRR using CO_2_ as the carbon source. As shown in Figure 4 a, the comparison of PCN and *o*‐PCN conclusively shows that the hydrophobic surface is highly effective in restraining the HER, elevating the selectivity (calculated based on electrons, for details see section 7 of methods) of the reduction towards carbon derivatives from 5.3 % to 84.2 %, while the generation rate for both is relatively low. After Pt loading, the generation rate of both H_2_ and carbon derivatives are massively increased, owing to the ability of Pt to collect electrons and to catalyze reduction reactions.[^1b^, ^2^, ^28^] For the catalyst with Pt loaded on the default hydrophilic surface (Pt/PCN), the HER is very fast, and most electrons are used for H_2_ generation, resulting in a low carbon derivative selectivity of only 2.5 %. However, on the hydrophobic surface, Pt is more effective in catalyzing carbon derivative generation instead of H_2_, indicating that the competitiveness of the CRR is remarkably enhanced. Electrons collected by Pt are more likely to be delivered to CO_2_, increasing its selectivity to 87.9 %, about 34 times higher than Pt/PCN. The generation rate of CO and CH_4_ can achieve 2.86 and 1.36 μmol h^−1^, respectively, with only 10 mg of the catalyst. Moreover, the relatively good photocatalytic stability of Pt/*o*‐PCN was demonstrated by a three‐run cycling test as monitored over the time (see Figure S5).^[30]^ The turnover numbers (TONs) of 14.458 for CO and CH_4_ were achieved over 9 hours of stable operation (see section 10 of methods), reflecting the catalytic nature of the reaction.^[31]^ To confirm that the carbon source of the resulting products is CO_2_ and HCO_3_^−^, isotope‐labeled CO_2_ was employed (see section 11 of methods),^[32]^ and carbon derivatives carrying the isotopes ^13^C and ^12^C were screened by a gas chromatography‐mass spectrometer (GC‐MS).[^30a^, ^33^] Results reveal that the mass‐charge ratios of 16 (*m*/*z=*16) and 28 (*m*/*z=*28) are ^12^CH_4_ and ^12^CO, respectively, generated from ^12^CO_2_,[^30a^, ^33^, ^34^] and the mass‐charge ratios of 17 (*m*/*z=*17) and 29 (*m*/*z=*29) can be assigned to ^13^CH_4_ and ^13^CO, respectively, produced from ^13^CO_2_,[^30a^, ^33^, ^34^] proving that the products indeed originate from CO_2_ reduction (see Figure S6). This conclusion can also be supported by the control experiments with or without CO_2_ (see Figure S7). The action spectra were also detected under the irradiation of different wavelengths (λ; see section 12 of methods and Table S1 and Figure S8),^[35]^ indicating that Pt/*o*‐PCN is more efficient under blue visible light (*λ*≈420 nm) and near ultraviolet light (*λ*≈380 nm). According to the action spectra and the conditions of incident irradiation, the apparent quantum efficiency (AQE) can be calculated (see section 13 of methods and Table S1 and Figure S8).[^27a^, ^36^] The highest AQE within the visible region is around 3.337 %, which is comparable to the carbon nitride based catalysts for CO and CH_4_ generation (generally 0.0086 % ∼ 5.70 %[^31c^, ^37^]). To further investigate the advantage of Pt as the active sites of reduction reaction, we also synthesized Cu‐ and Pd‐loaded *o*‐PCN (denoted as Cu/*o*‐PCN and Pd/*o*‐PCN, respectively; see section 5 of methods and Figure S9 a,b). Results show that over both Cu/*o*‐PCN and Pd/*o*‐PCN, the generation rates of CO and CH_4_ are lower than with Pt/*o*‐PCN (see Figure S9 c), indicating the fine performance of Pt for this reduction reaction. The selectivity and distribution of products can be tunable by changing the cocatalysts from Pt to either Cu or Pd, but the selectivity of derivatives still remain at a relatively high value (see Figure S9 c), which may be attributed to the hydrophobic surface.

**Figure 4:**
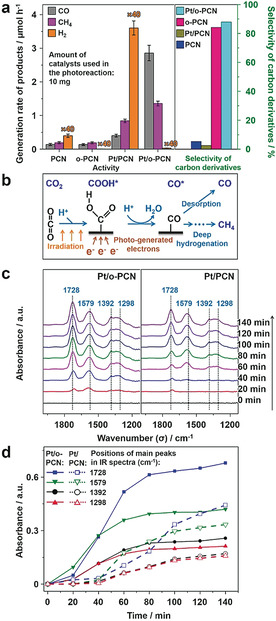
a) The activity of the photocatalytic CRR and selectivity towards carbon derivatives. The amount of every catalyst used in the photoreaction is 10 mg. The note ×40 means the value of this column shown here is reduced by 40 times. b) The general reaction process of CRR. c) in situ IR spectra of Pt/PCN and Pt/*o*‐PCN. d) The change of absorbance intensity of main peaks belonging to *COOH in the in situ IR spectra over time.

The mechanism behind this enhancement can be attributed to the increased concentration of CO_2_ adsorbed onto the hydrophobic surface, and can also be proved by the in situ infrared (IR) spectrum (Figure 4 c).[^2^, ^38^] Under irradiation, the photogenerated electrons are collected by Pt particles to create a negatively charged active sites.^[18a]^ The activated CO_2_ molecules are adsorbed on it in the form of carboxy group (*COOH; Figure 4 b),[^2^, ^38^] which can be detected by in situ IR (details see section 8 of methods). The peaks at 1728, 1579, 1392, and 1298 can be attributed to the stretching vibration of the carboxy group in carboxylic acids (*ν*(C=O)), the antisymmetric telescopic vibration of OCO (*ν*_as_(OCO)), the symmetrical stretching vibration of COO^−^ (*ν*_s_(COO^−^)), and the stretching vibration of carboxylic acid hydroxylgroup (*ν*(C−OH)), respectively, all belonging to *COOH.[^38b^, ^38c^, ^39^] Because no formate species can be detected in the active test, we can deduce that *COOH is more likely to be further dehydrated to form CO*, and finally, generate CO and CH_4_ (Figure 4 b).[^2^, ^38^, ^40^] Results (Figure 4 c,d) show that the concentration of the *COOH species increases rapidly at the beginning of the reaction, and then remains constant over time, and is due to the equilibrium between CO_2_ adsorption and *COOH consumption by reduction (to form *CO in subsequent process as shown in the Figure 4 b).[^2^, ^38^] The Pt/*o*‐PCN shows a faster increase of absorbance and a higher equilibrium value of the characteristic peaks belonging to *COOH compared to Pt/PCN (Figure 4 c,d), indicating that effective CO_2_ adsorption is indeed enhanced in the fluorophilic patches, which then spill over to the Pt‐particles nearby. Because the adsorption of CO_2_ in the form of COOH* is a result of the effective mass transfer of the gas phase to the surface, the rate of increase of the main IR signal peaks of COOH* may be used as the quantitative parameter to reflect the effective mass transfer (see Figure S10). The rate of increase over the hydrophobic surfaces is about three times more than the hydrophilic surface (see Figure S10), indicating an enhanced effective mass transfer of CO_2_ with the hydrophobic catalyst.

Based on all the evidence above, it is reasonable to propose a model for the CO_2_ photoreduction with the three‐phase photocatalyst, as shown in Figure 1. Electrons and holes are generated under irradiation, and then the holes are consumed by the sacrificial agent, while electrons are trapped by Pt particles because of the large work function of Pt. The hydrophobic patches are adverse water wetting and promote increased gas adsorption on the surface, resulting in a three‐phase contact interface with highly concentrated CO_2_, which then coats the negatively charged Pt particles with carboxylates. In this situation, the mass‐transfer limitation is overcome, and thus more photogenerated electrons are delivered to CO_2_ to generate carbon derivatives, while the overall CRR is enhanced.

In conclusion, this paper introduces a PCN‐based photocatalyst with fluorophilic polymer surface patches, allowing the efficient three‐phase contact of CO_2_ (gas), H_2_O (liquid), and catalyst (solid). Thus, a high concentration of CO_2_ molecules are able to reach the catalyst surface directly. This modification breaks the mass‐transfer limitation of CO_2_, inhibits the HER, and enhances the CRR at the same time. Even when Pt, one of the most efficient electron collection agents and HER promotion cocatalysts, is used, our three‐phase photocatalysts still exhibits a high CRR selectivity of up to 87.9 %, about 34 times higher than the commonly used hydrophilic catalysts made of similar materials. Additionally, the three‐phase photocatalysis method is portable and reliable, and can provide inspiration for the design if photocatalysts for CO_2_ reduction, or other reactions, such as photocatalytic nitrogen fixation, which require a three‐phase contact.

## Conflict of interest

The authors declare no conflict of interest.

## Supporting information

As a service to our authors and readers, this journal provides supporting information supplied by the authors. Such materials are peer reviewed and may be re‐organized for online delivery, but are not copy‐edited or typeset. Technical support issues arising from supporting information (other than missing files) should be addressed to the authors.

Supplementary
