# Supplementary material for: Three‐Phase Photocatalysis for the Enhanced Selectivity and Activity of CO2 Reduction on a Hydrophobic Surface
Source: Angew Chem Int Ed Engl. 2019 Sep 4;58(41):14549–55. doi: 10.1002/anie.201908058 (PMC7687246; doi:10.1002/anie.201908058)
Supplement: Supplementary file 1 — Supplementary [file ANIE-58-14549-s001.pdf]

Angewandte Chemie
Eine Zeitschrift der Gesellschaft Deutscher Chemiker

Supporting Information

Three-Phase Photocatalysis for the Enhanced Selectivity and Activity of CO_2 Reduction on a Hydrophobic Surface

Ang Li, Qian Cao, Guangye Zhou, Bernhard V. K. J. Schmidt, Wenjin Zhu, Xintong Yuan, Hailing Huo, Jinlong Gong, and Markus Antonietti**

anie_201908058_sm_miscellaneous_information.pdf

Experimental Procedures

Materials

Acetone, Al_2O_3 basic, 2-bromopropionyl bromide (98%), cyanuric acid (98%), ethanol, ethyl acetate (99.8 %), hexane (95%), melamine (99%), *N,N,N',N",N"*-pentamethyldiethylenetriamine (PMDETA, ≥98%), 1*H*,1*H*,2*H*,2*H*-perfluorodecanethiol (pFDe, ≥97%), pyridine (99.8%), silica gel (for column chromatography), tetrahydrofuran (THF, ≥99.9%, inhibitor-free) and lithium hydroxide (LiOH, 99.9%), Palladium(II) acetylacetonate (Pd(acac)$_2$, 99%), Copper(II) acetylacetonate (Cu(acac)$_2$, ≥99%) and ^{13}C labeled sodium bicarbonate ($NaH^{13}CO_3$, 98 atom% ^{13}C) were purchased from Sigma Aldrich. Hydrochloric acid (1 mol L^{-1}) was purchased from Merck KGaA. Platinum(II) bis(acetylacetonate) (Pt(acac)$_2$) was purchased from Tokyo Chemical Industry UK Ltd. Na_2S (≥98.0) and Na_2SO_3 (≥97.0) were purchased from Tianjin Fuyu Fine Chemical Co. Ltd. $KHCO_3$ (99.5%) was purchased from Macklin. Deionized water (H_2O, 18.25 MΩ·cm) supplied by an UP Water Purification System was used in the whole experimental processes. Copper (I) bromide (Cu(I)Br, 98%, Sigma Aldrich) was purified by stirring in glacial acetic acid overnight, filtered, and washing with absolute ethanol. Glycidyl methacrylate (GMA, 97%, Sigma Aldrich) was passed through a basic alumina column prior to use. All other chemicals were obtained from commercial suppliers and used without further purification.

Characterization

Crystalline structures were evaluated by X-ray diffraction (XRD) analysis using a Bruker D8 Advance instrument with Cu Kα radiation. The Transmission Electron Microscope (TEM) is a double-corrected Jeol ARM200F, equipped with a cold field emission gun. The acceleration voltage was set to 200kV and the emission was put to 10 μA. Room temperature photoluminescence (PL) spectra were monitored at a Perkin Elmer Luminesence Spectrometer (LS 50 B) with excitation wavelength of 350 nm. The specific surface areas were calculated from the isotherms using the BET method. The pore distribution and the cumulative volumes of pores were obtained by the BJH method from the desorption branch of the nitrogen adsorption isotherms. Inductively Coupled Plasma Optical Emission Spectrometer (ICP-OES) was performed on a Vista MPX ICP. *in situ* infrared (IR) spectrum was performed on Thermo Fisher Nicolet iS50. X-ray photoelectron spectroscopy (XPS) was performed under ultrahigh vacuum (<10-6 Pa) on a Kratos XSAM 800 spectrometer with Mg Kα X-ray source (E = 1253.6 eV). Gas chromatography-mass spectrometer was recorded by HIDEN QIC-20 mass spectrometer. The optical properties of the powders were examined by a Shimadzu UV 2600 instrument. Here we use the absorbance pattern to detect the light absorption within the whole range of UV and visible light. For solid samples, the light absorbance is detected based on diffuse reflection light. $BaSO_4$ is used as the reference sample. The reflection light when $BaSO_4$ is used is considered to have an absorbance of 0, which is used as the reference light. The absorbance is the part of light less than reference light. The value of the absorbance is calculated by the built-in program of the instrument.

Methods

Section 1. Synthesis of polymeric carbon nitride (PCN). The carbon nitride used in present work was synthesized via cyanuric acid and melamine as precursors. A 1:1 molar ratio of cyanuric acid and melamine were mixed in water and shaken for 24 hours at ambient temperature. Then the obtained milky suspension was centrifuged and washed with water three times, and dried at 50 °C under vacuum. Afterwards the precursor powder was annealed in capped crucibles at 550 °C for 4 h at a heating rate of 2.3 °C/min in nitrogen atmosphere. After cooling down the yellow carbon nitride powder was collected without any further treatment.[1]

Section 2. Modification the surface to synthesis hydrophobic PCN (o-PCN). The o-PCN was obtained through 3 steps.[2] (1) Synthesis of ene-PGMA. To obtain PGMA with double bond end modification, an ene-initiator (undecenyl 2-bromopropionate) was synthesized firstly according to the literature.[3] Then GMA was polymerized using atom transfer reaction polymerization (ATRP) with Cu(I)Br as catalyst, PMDETA as ligand and ene-initiator. Specifically, Cu (I) Br (15 mg) was added to a 25 mL round bottom flask in an ice bath, the flask was sealed with a rubber septum and flushed with Argon for 30 min. Subsequently, deoxygenated GMA (5.4 g) and PMDETA (17 mg) were added via syringe. After the solution turned light green, the ene-initiator (64 mg) was added via syringe. The mixture was stirred at 25 °C for 30 min. Thus, PGMA with ene-functionalization was prepared (ene-PGMA, M_n=25.9 kg/mol). (2) Grafting of ene-PGMA onto PCN was realized by mixing 300 mg ene-PGMA with 30 mg PCN in 15 mL THF in a round bottom flask, the flask was sealed with a rubber septum and flushed with Argon for 30 min. The mixture was put between two 50 W LED daylight sources (20 cm distance between light sources) to initiate the grafting. After 48 h reaction, the mixture was washed 2 times with THF and dried under vacuum, to obtain ene-PGMA/PCN. (3) Hydrophobic modification of ene-PGMA/PCN. In a 15 mL glass vial, 2 mL THF, 7 mg of ene-PGMA/PCN and 300 μL pFDe were mixed and cooled with an ice bath. 3 mg LiOH was added slowly, the cooling was removed and the resulting reaction mixture was stirred at ambient temperature for 1 hour. Afterwards, the mixture was diluted with THF and washed 3 times with 3 mL THF and centrifuged. The precipitate was dried under vacuum to obtain o-PCN.

Section 3. Pt loading on PCN to synthesize Pt/PCN. 0.3 g PCN was dispersed in an aqueous solution containing 20 mL H_2O and 10 mL ethanol under sonication. Then 40 μL H_2PtCl_6 solution (the concertation of the H_2PtCl_6 solution is 8 wt%) was added with sonication. After stirring for 30 min, the mixture was moved to a sealed transparent container which was set under a pressure of 0.13 bar. Subsequently, the mixture under 0.13 bar was irradiated under visible light supplied by the visible light source for 18 h. Subsequently, the as-prepared sample was separated by centrifugation and washed three times with H_2O. Then the samples were vacuum dried at 80 °C overnight to finally form Pt/PCN as a powder.

WILEY-VCH

Section 4. Pt loading on o-PCN to synthesize Pt/o-PCN. 0.3 g o-PCN was dispersed in the mixture of 20 mL acetone and 10 mL ethanol under sonication. Then 0.03 g Pt(acac)$_2$ was added to the mixture under sonication. After stirring for 30 min, the mixture was moved to a sealed transparent container which was set under a pressure of 0.13 bar. Subsequently, the mixture under 0.13 bar was irradiated under visible light supplied by the visible light source for 18 h. Then the as-prepared sample was separated by centrifugation and washed three times with acetone. Then the samples were vacuum dried at 80 °C overnight to finally form Pt/o-PCN as a powder.

Section 5. Cu and Pd loading on o-PCN to synthesize Cu/o-PCN and Pd/o-PCN. 0.3 g o-PCN was firstly dispersed in the mixture of 20 mL acetone and 10 mL ethanol under sonication. Then 0.020 g Cu(acac)$_2$ or 0.023 g Pd(acac)$_2$ was added to the mixture under sonication. After stirring for 30 min, the mixture was moved to a sealed transparent container which was set under a pressure of 0.13 bar. Subsequently, the mixture under 0.13 bar was irradiated under visible light supplied by the visible light source for 18 h. Then the as-prepared sample was separated by centrifugation and washed three times with acetone. Then the samples were vacuum dried at 80 °C overnight to finally form Cu/o-PCN or Pd/o-PCN as a powder. The TEM and HRTEM images of Cu/o-PCN and Pd/o-PCN are shown in Figure S9, in which the Cu or Pd particles loaded on o-PCN can be clearly observed, and the lattice spacing can prove the composition.

Section 6. Photocatalytic CO_2 reduction. The home-made photocatalytic reactor consisted of a sealed chamber, an embedded window made by quartz glass and a liquid sampling port sealed by silicone pad. The reactor was connected to a gas circulation system with a ten-port valve (VICI) for on-line sampling to a gas chromatograph (Ruimin GC 2060, Shanghai). The gas circulation system was primarily made of stainless steel tubing and a home-made gas pump for the circulation of the gas. A mechanical pump was connected into the system to exhaust the carrier gas of the gas chromatograph when switch back the ten-port value. A pressure gauge was also connected into the system to monitor the gas pressure. The total volume of the gas in the circulation system after filling the reactor with solvent was 80 mL.

At the start of this reaction, 0.01 g catalysts were mixed with 40 mL $KHCO_3$ (0.1 M), Na_2S (0.1 M) and Na_2SO_3 (0.1 M) aqueous solution in the reactor. $KHCO_3$ is used to solution to enhance the solubility of CO_2 during photocatalytic reactions. Upon the introduction of CO_2, a chemical equilibrium ($HCO_3^- + H_2O \rightleftharpoons H_2CO_3 + OH^- \rightleftharpoons CO_2{+}H_2O{+}OH^-$) will be established.[4] With the consumption of CO_2, this chemical equilibrium will shift right, while if CO_2 is introduced again, this chemical equilibrium will shift left.[4-5] Thus, even without CO_2, there is also a few of CO_2 molecules existing in $KHCO_3$ solution, which can be used to produce carbon derivatives (Figure S7). However, without CO_2, the photoreaction is unsustainable, because CO_2 and $KHCO_3$ in the solution will be used up soon. This chemical equilibrium can be proved by the isotope experiment reported by Dunwell et al.[5] They use $^{13}CO_2$ purged $NaH^{12}CO_3$ for the reaction of CO_2 reduction, and results showed that ^{13}C atoms exist in both dissolved CO_2 and $KHCO_3$: $^{13}CO_{2\,(aq)} + H^{12}CO_3^- \rightleftharpoons {}^{12}CO_{2\,(aq)} + H^{13}CO_3^-$.[5]

The system was evacuated to remove air firstly. Subsequently, the suspension in the reactor was purged with CO_2 (≥ 99.995%) for 1 h to achieve CO_2 saturation and the initial CO_2 pressure was kept atmospheric. There was no more CO_2 purged into the closed system during the reaction. The gases in the closed circulation system were continuously circulated through the suspension for the entire reaction period. The incident light source was supplied by a 300 W xenon lamp and a visible light filter (Beijing PerfectLight Co.), which can provide the visible light with the wavelength longer than 400 nm (λ > 400 nm). After the reaction of 1 h, the gas and liquid products was detected by the analysis system.

The analysis of the gaseous reaction mixtures containing CO, CH_4, H_2, was carried out using a gas chromatograph, which was equipped with a TCD, FID and a methanizer which contained a Ni catalyst. Argon (≥ 99.999%) was used as the carrier gas. The back channel of GC was equipped with two packed columns, TDX-01 and Molsieve 5 Å, and two gas switch valves. During the analysis, 1.0 mL of gas sample in the sample loop of ten-port value was introduced to the TDX-01 column where CO_2 was separated from the other gases due to its longer retention time. The rest of the gases after the TDX-01 column was further separated by the Molsieve 5 Å column. The gas product of H_2 was detected by TCD and CH_4, CO were further detected by FID with higher sensitivities. The role of methanizer was to convert CO to CH_4 for FID analysis. The liquid product of CH_3OH was injected into HP-5 capillary column (30 m × 0.32 mm ID × 0.5 μm, Agilent) and detected by FID after the reaction. No CH_3OH can be detected in this work. Other liquid products such as HCOOH and HCHO possibly formed during the reaction were also analyzed by high-performance liquid chromatography (HPLC 7080B, Agilent Co., USA), but no such products were detected under our reaction conditions. Cycling test was operated using the same method, and each cycle continued 3 h. after each cycle, the catalysts were separated by centrifugation, dried under vacuum at 80 °C for 6 h and then reused in the next cycle.

Section 7. Calculation of selectivity of carbon derivatives. The selectivity of CO_2 reduction to carbon derivatives (CO, CH_4) on an electron basis ($8e^-$ for CH_4, $2e^-$ for CO and H_2[6]) has been calculated using the following equation:[7]

Selectivity of carbon derivatives (%) $= [2n(CO) + 8n(CH_4)] / [2n(CO) + 8n(CH_4) + 2n(H_2)] \times 100\%$

Where $n(CO)$, $n(CH_4)$ and $n(H_2)$ are the amounts (moles) of CO, CH_4, and H_2 formed within a certain period of time.

Section 8. *in situ* IR test. A diffuse reflection *in situ* IR was used to investigate the effective CO_2 adsorption on the surface. Samples are loaded in the sample holder with a flat surface. A cover was fixed on the sample holder to form a reaction space, which was then cleaned by argon. Then the IR data was collected as background. Subsequently, visible light was introduced to the reaction space by observation window, and CO_2 was introduced through container filled with water which make the gas bring water into the reaction space. Then IR data was collected every 20 min, and the change based on IR can be recorded.

Section 9. Stability of the polymer-modified surface during visible irradiation and during the reaction. The polymer modified surface of o-PCN is very stable under visible light irradiation and during the photoreduction, which can be proved by several evidences. (1) As shown in insert of Figure S7, after the photoreaction under visible light, the contact angle of water on o-PCN is 91.5°, which is very close to that before photoreaction (89.7°), almost remaining unchanged. Because the hydrophobic properties of o-PCN are provided by the polymer, it can be deduced that the polymers remain stable under visible irradiation and during the photoreduction. (2) It has been explained in details in section 6 of *methods* and Figure S7 that the carbon source of carbon derivatives is CO_2, which means the carbon skeleton of polymer did not react during photoreaction. (3) As described in section 2 of *methods*, the polymer was connected to PCN by visible light with 48 h, and after that the hydrophobicity of surface was greatly improved. It can be deduced that if the polymer is unstable under visible light, it will be decomposed totally in 48 h and would not improve the hydrophobicity of the surface.

Section 10. The calculation of turnover number (TON) over Pt/o-PCN. The turnover number (TON) was calculated using the following equation:[8]

$$\text{TON}=\frac{\text{moles of products evolved}}{\text{moles of active components on photocatalyst}} \qquad (1)$$

Moles of products evolved: In this work, only CO and CH_4 can be detected as the products of CO_2 reduction. We detected the amount of both CO and CH_4 base on the method described in section 6 of *methods* in the SI. The Pt/o-PCN can remain relatively stable during 9 h. After the reaction of 9 h with 0.01 g Pt/o-PCN, the amounts of CO and CH_4 were detected to be 12.751 and 6.594 μmol, respectively.

Moles of active components on photocatalyst: the loading amount of Pt was determined to be 2.61 wt% by the test of ICP. Thus, the moles of active components is (0.01 g × 2.61%) / 195.1 g mol^{-1} = 1.338×10^{-6} mol = 1.338 μmol.

Thus, TON = (12.751 + 6.594) μmol / 1.338 μmol = 14.458. The TON value is obviously more than 1, reflecting the catalytic nature of the reaction.[9]

Section 11. Investigation of carbon source with isotope labeled CO_2. The reaction system of the isotope experiment is the sealed circulatory system mentioned in section 6 of *methods* in SI. 1 g $NaH^{13}CO_3$ (98 atom% ^{13}C, Sigma Aldrich) was added into 32.2 mL Na_2S (0.1 M) and Na_2SO_3 (0.1 M) aqueous solution in the reactor to provide $H^{13}CO_3^-$. Then after adding 0.01 g Pt/o-PCN, the system was sealed. The system was evacuated to remove air firstly, and then the suspension in the reactor was purged with argon for 1 h to remove air. Then 7.8 mL hydrochloric acid (1 M) was injected through the liquid sampling port with silica gel pad for the *in situ* generation of $^{13}CO_2$ *via* the reaction $H^+ + H^{13}CO_3^- = H_2O + {}^{13}CO_2$. According to the stoichiometric ratio, the amount of hydrochloric acid added can ensure the remaining of 4 mmol $H^{13}CO_3^-$ in these 40 mL mixture, making the concentration of $H^{13}CO_3^-$ to be 0.1 M. Then after a circulation for 1 h, visible light was provided for the photocatalytic reaction. After the reaction of 5 h, the gas sample was detected by a gas chromatography-mass spectrometer (GC-MS) instrument.

The gas chromatograph (GC) is the same one that we used to detect reaction activity (described in section 6 of *methods* in SI) with the columns of TDX-01 and Molsieve 5 Å. The difference is that the gas products separated by GC was introduced into a mass spectrometer (HIDEN QIC-20) instead of Flame Ionization Detector (FID). We monitored the characteristic mass-charge ratios (m/z) of 28, 29, 16 and 17 to detect the ^{12}CO, ^{13}CO, $^{12}CH_4$ and $^{13}CH_4$ in the product, respectively, and results are shown in Figure S6. According to the results of GC recorded by FID in the former experiment when detecting activity (section 6 of *methods* in SI), the retention time of CO and CH_4 is 4.990 and 9.234 min, respectively. Thus, because the same GC was used for GC-MS, the signal appeared in similar retention recorded by MS were the time is reasonable to be recognized as the signal of CO and CH_4. The retention time of carbon derivatives with ^{13}C is slightly larger than that with ^{12}C, which also have been observed by other researchers.[10] Figure S6b shows that ^{12}CO can be detected when $^{12}CO_2$ and $H^{12}CO_3^-$ are provided, and ^{13}CO can be detected when $^{13}CO_2$ and $H^{13}CO_3^-$ are used as the carbon source.[11] Figure S6c shows that $^{12}CH_4$ can be detected when $^{12}CO_2$ and $H^{12}CO_3^-$ are provided, and $^{13}CH_4$ can be detected when $^{13}CO_2$ and $H^{13}CO_3^-$ are used as the carbon source.[10] These results proved that the photocatalytic products indeed originate from CO_2 reduction.[10]

Section 12. The action spectra of photocatalytic CO_2 reduction reaction (CRR). The action spectra is a graph of the rate of an activity plotted against the wavelength of light.[12] To detect the activity of CRR with Pt/o-PCN under the irradiations with different wavelength, we use a similar method which was described in section 6 of *methods* in the SI. The only difference is the incident light was changed from visible light (λ > 400 nm) to monochromatic light with a specific wavelengths, which were provided by a 300 W xenon lamp (Beijing PerfectLight Co.) and various bandpass filters (Beijing China Education Au-light Co., Ltd). The CRR activities under monochromatic irradiations with wavelengths of 365, 380, 420, 435, 525 and 700 nm are listed in Table S1, According to which we can get the action spectra (Figure S8).

Section 13. Apparent quantum efficiency (AQE) for photocatalytic CRR. The AQE is defined by the equation:[13]

$$\varphi_x=\frac{\mp(d[x]/dt)}{d[h\nu]_{inc}/dt} \qquad (2)$$

where d[x]/dt is the rate of change of the concentration of the reactant (or product) and $d[h\nu]_{inc}/dt$ is the total optical power impinging on the sample. Generally, for the convenient of measurement and calculation, researchers usually use the integral form of equation (2):[14]

$$\mathrm{AQE}(\%)=\frac{\text{number of the electrons taking part in reaction}}{\text{number of incident photons}}\times 100\% \qquad (3)$$

The number of electrons taking part in the reaction is calculated according to the action spectra, which was described in section 12 of *methods* in the SI. In this work, the products of CRR are only CO and CH_4. Thus, the AQE of CRR should be calculated according to the action spectra of CO and CH_4. Normally, two electrons are needed to get one molecule of CO, and eight electrons are needed to get one molecule of CH_4. Thus, equation (3) can be transformed into:[14b]

$$\mathrm{AQE}(\%)=\frac{2\times\text{number of evolved CO molecules}+8\times\text{number of evolved CH}_4\text{ molecules}}{\text{number of incident photons}}\times 100\% \qquad (4)$$

The numerator and denominator of equation (4) are divided by unit time simultaneously, we can get:[15]

$$\mathrm{AQE}(\%)=\frac{(2\times\text{number of evolved CO molecules}+8\times\text{number of evolved CH}_4\text{ molecules})/\text{unit time}}{\text{number of incident photons/unit time}}\times 100\% \qquad (5)$$

We denote the numerator of equation (5) as N_e, and the denominator of equation (5) as N_p. Thus, the N_e means the amounts of electrons taking part in CRR during the unit time, and N_p means the amounts of incident photons during the unit time, and equation (5) can be written as:[15]

$$\mathrm{AQE}(\%)=\frac{N_e}{N_p}\times 100\% \qquad (6)$$

Thus, we can calculate the AQE as long as we can get the value of N_e and N_p under the incident light with a specific wavelength. Here we demonstrate the process with the example of AQE calculation under irradiation with the wavelength of 420 nm.

N_e: The amount of CO and CH_4 generated by unit catalyst at the unit time was detected under the irradiation with the wavelength of 420 nm (as described in section 12 of *methods* in the SI). As shown in Table S1, the generation rate of CO and CH_4 under 420 nm is 0.531 and 0.229 μmol h^{-1}, which can be transferred to be 1.475×10^{-10} and 6.361×10^{-11} mol s^{-1}, respectively. Thus, $N_e = 2 \times 1.475 \times 10^{-10} + 8 \times 6.361 \times 10^{-11} = 8.039 \times 10^{-10}$ mol s^{-1}.

N_p: The energy of single photon (denoted as E_s) at λ = 420 nm can be calculated to be 4.736×10^{-19} J according to $E_s = (hc) / \lambda$, where h and c are the Planck constant (6.626×10^{-34} J·s) and the speed of light (2.998×10^{8} m s^{-1}), respectively. The light intensity (denoted as I_{in}) was measured to be 0.35 mW cm^{-2} by an irradiatometer (Photoelectric Instrument Factory of Beijing Normal University). The diameter (denoted as d) of illumination window of the reactor was measured to be 5 cm, and thus the irradiation area (denoted as S_{in}) can be calculated to be 19.63 cm^2 according to the equation $S_{in}=\pi \times (d/2)^2$. Thus, the N_p can be calculated by divide the total energy provided in unit time by the energy of single photon: $N_p = (I_{in} \times S_{in}) / E_s = 2.408 \times 10^{-08}$ mol s^{-1}. [16]

Thus, according to equation (6), AQE (%) = $N_e / N_p \times$ 100%= 8.039×10^{-10} mol s^{-1} / 2.408×10^{-08} mol s^{-1} × 100% = 3.337%.

In addition, the AQE of CRR at λ = 365, 380, 435, 525 and 700 nm can be calculated by the same process. Results are listed in Table S1, according to which we can get the AQE spectra as shown in Figure S8.

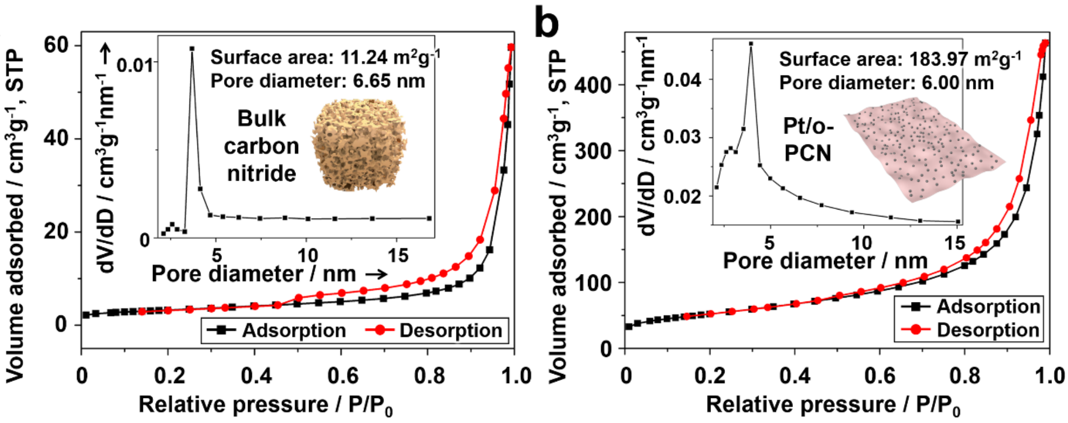

Figure S1. Nitrogen adsorption isotherms and pore size distribution (inset) of a) bulk carbon nitride and b) Pt/o-PCN.

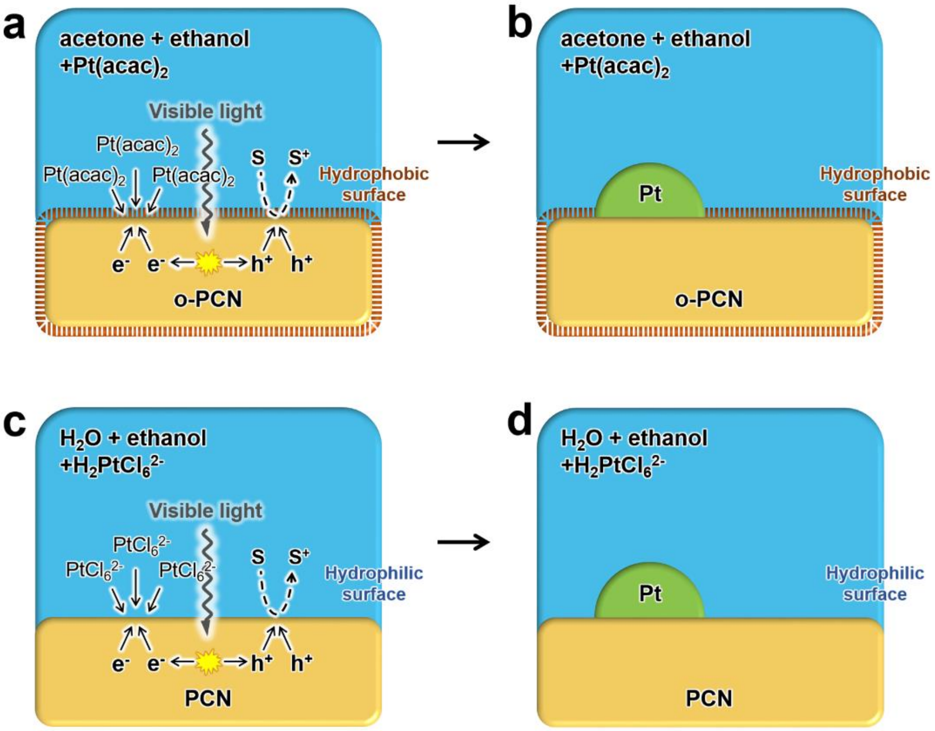

Figure S2. The mechanism of *in situ* photo-loading method to synthesis Pt/o-PCN and Pt/PCN. a) The progress of photo-loading to load Pt particles on o-PCN. S represents the hole sacrificial agent, and S^+ represents the sacrificial agent which are already oxidized by holes. Here ethanol is used as the sacrificial agent. b) The formation of Pt/o-PCN. c) The progress of photo-loading to load Pt particles on PCN. S represents the hole sacrificial agent, and S^+ represents the sacrificial agent which are already oxidized by holes. Here ethanol is used as the sacrificial agent. d) The formation of Pt/PCN.

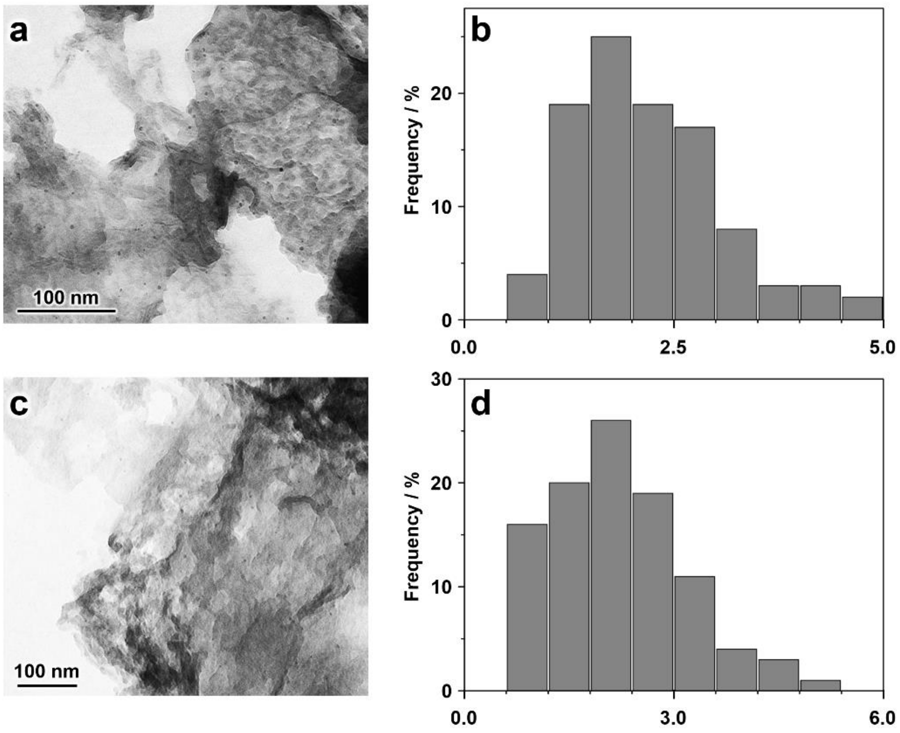

Figure S3. a) TEM images of Pt/PCN. b) Pt particle size statistics of Pt/PCN based on image a. Average particle size: 2.18 nm. c) TEM images of Pt/o-PCN. d) Pt particle size statistics of Pt/o-PCN based on image c. Average particle size: 2.21 nm. Pt Particles taking part in statistics are selected randomly.

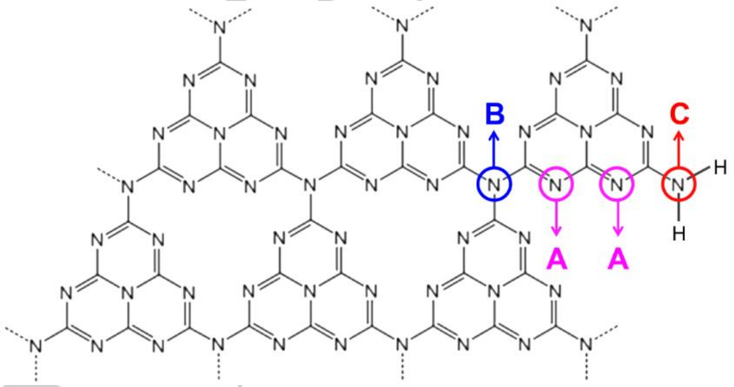

Figure S4. Nitrogen atoms in different chemical surroundings with different bonding mode. A: C-N=C. B: N-$(C)_3$. C: C-N-H.

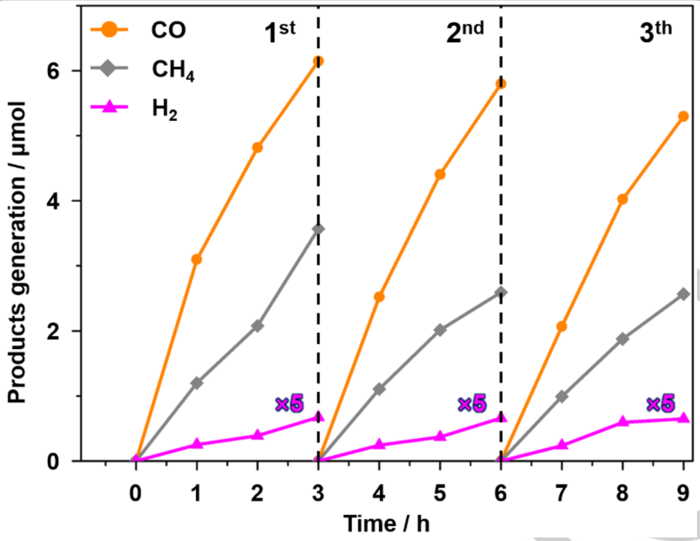

Figure S5. Stability along with time and recycling test of Pt/o-PCN.

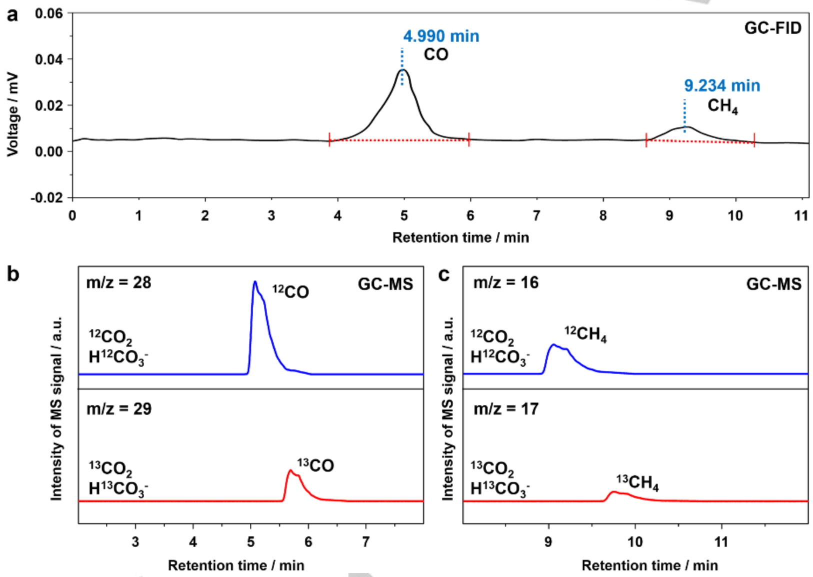

Figure S6. Signal of GC-FID and GC-MS. a) the raw data of the signal of GC-FID which was used to investigation the activity using the method described in section 6 of *methods* in the SI. b) The GC-MS signal monitored by m/z = 28 and m/z = 29 for the detection of ^{12}CO and ^{13}CO, respectively.[11] c) The GC-MS signal monitored by m/z = 16 and m/z = 17 for the detection of $^{12}CH_4$ and $^{13}CH_4$, respectively.[10]

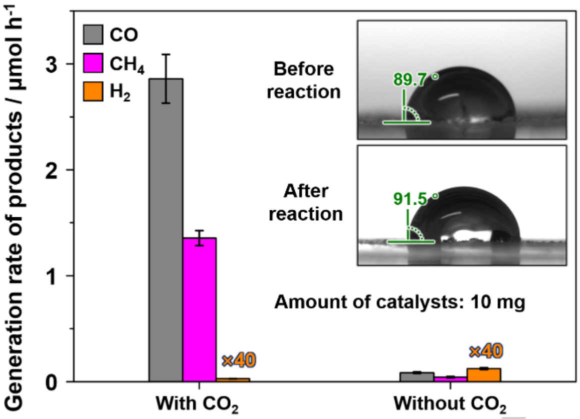

Figure S7. To investigate the carbon source of CRR with o-PCN, control experiment was operated by replacing CO_2 to N_2. Result shown that without CO_2 there are almost no carbon derivatives can be detected. A few carbon derivatives in products can be attribute to the $KHCO_3$ solution, which is described in detail in section 6 of methods. Insert: Photographs of contact angles of water on o-PCN before and after photoreaction.

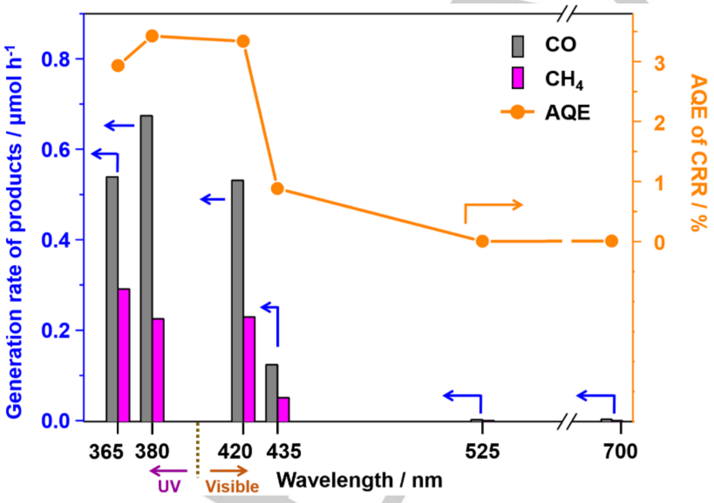

Figure S8. The action spectra and AQE spectra with the catalysts of Pt/o-PCN. This figure is constructed according to the data shown in Table S1. Under the irradiation with different wavelengths, the ration of CO and CH_4 is variable. The highest AQE within the visible region is 3.337, which is achieved under the irradiation of 420 nm.

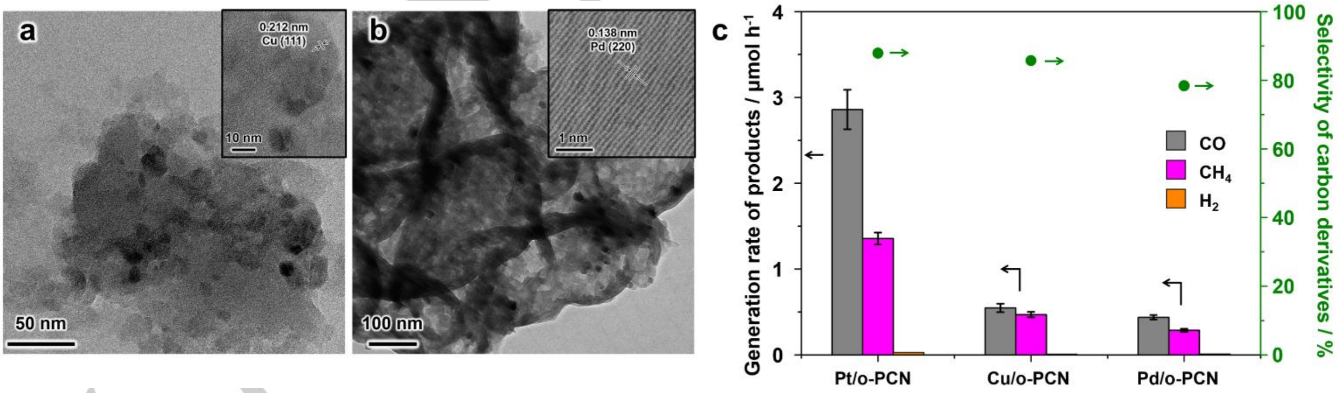

Figure S9. The TEM and high-resolution TEM (HRTEM) images HRTEM images of Cu/o-PCN and Pd/o-PCN and their CO_2 photoreduction activities. a) The TEM images of Cu/o-PCN. Insert: HRTEM image of Cu particles. The lattice spacing is 0.212 nm, which is in good agreement with the (111) planes of Cu. b) the TEM images of Pd/o-PCN. Insert: HRTEM image of Pd particles. The lattice spacing is 0.138 nm, which is in good agreement with the (220) planes of Pd. c) the CO_2 reduction activities and selectivity over Cu/o-PCN and Pd/o-PCN, and the comparison with Pt/o-PCN. Only CO and CH_4 can be detected as the products of CO_2 reduction.

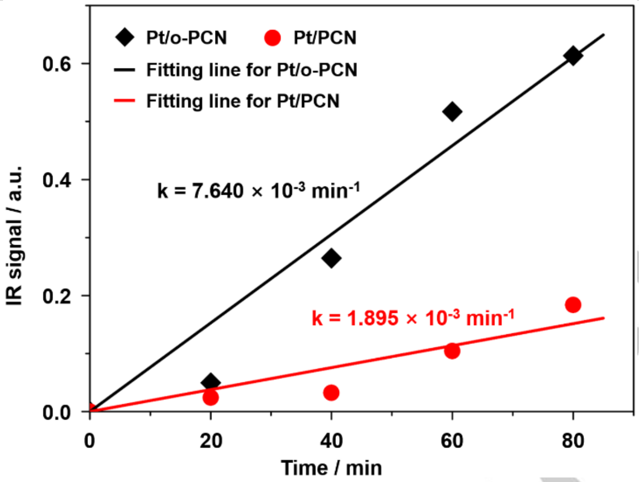

Figure S10. The quantitative analysis of effective mass transfer. Because the adsorption of CO_2 as the form of COOH* is a result of the effective mass transfer of gas phase to the surface, the rate of increase of the main IR signal peaks of COOH* may be used as the quantitative parameter to reflect the effective mass transfer. The main IR peak of COOH* is the one at the wavenumber of 1728 cm^{-1} (Figure 4d). The average increasing rates can be calculated based on the slope of fitting lines, which determined to be 7.040 × 10^{-3} min^{-1} and 1.859 × 10^{-3} min^{-1} for Pt/o-PCN and Pt/PCN, respectively.

Table S1. The data of action spectra and AQE under incident light with different wavelengths.[a]

Wavelength / nm	Generation rate /μmol h^{-1}		N_e[b] / mol s^{-1}	E_s[c] / J	I_{in}[d] / mW cm^{-2}	N_p[e] / mol s^{-1}	AQE[f] / %
	CO	CH_4					
365	0.539	0.291	9.461 × 10^{-10}	5.449 × 10^{-19}	0.54	3.229 × 10^{-08}	2.930
380	0.674	0.225	8.744 × 10^{-10}	5.234 × 10^{-19}	0.41	2.553 × 10^{-08}	3.426
420	0.531	0.229	8.039 × 10^{-10}	4.736 × 10^{-19}	0.36	2.408 × 10^{-08}	3.337
435	0.124	0.051	1.822 × 10^{-10}	4.572 × 10^{-19}	0.29	2.067 × 10^{-08}	0.882
525	0.002	0	1.111 × 10^{-12}	3.789 × 10^{-19}	0.38	3.269 × 10^{-08}	0.003
700	0.003	0	1.666 × 10^{-12}	2.841 × 10^{-19}	0.19	2.179 × 10^{-08}	0.008

[a] the action spectra and AQE are investigated using the target catalyst Pt/o-PCN. Incident light of different wavelengths was provided by a 300 W xenon lamp (Beijing PerfectLight Co.) and various bandpass filters (Beijing China Education Au-light Co., Ltd). [b] N_e means the amounts of electrons taking part in CRR during the unit time. [c] E_s means the energy of a single photon, which is calculated according to $E_s = (hc) / \lambda$, in which the h, c and λ are the Planck constant, the speed of light and the wavelength of the photon. [d] I_{in} means the intensity of incident light, which is measured by an irradiatometer (Photoelectric Instrument Factory of Beijing Normal University). [e] N_p means the amounts of incident photons during the unit time. [f] AQE was calculated according to the equation with integral form AQE = N_e / N_p.

References

[1] M. Shalom, S. Inal, C. Fettkenhauer, D. Neher, M. Antonietti, *J. Am. Chem. Soc.* **2013**, *135*, 7118-7121.
[2] Q. Cao, B. Kumru, M. Antonietti, B. V. K. J. Schmidt, *Macromolecules* **2019**, DOI: 10.1021/acs.macromol.1029b00894.
[3] a) K. Matyjaszewski, P. J. Miller, N. Shukla, B. Immaraporn, A. Gelman, B. B. Luokala, T. M. Siclovan, G. Kickelbick, T. Vallant, H. Hoffmann, T. Pakula, *Macromolecules* **1999**, *32*, 8716-8724; b) S. De, A. Khan, *Chem. Commun.* **2012**, *48*, 3130-3132.
[4] S. Zhu, B. Jiang, W. B. Cai, M. Shao, *J. Am. Chem. Soc.* **2017**, *139*, 15664-15667.
[5] M. Dunwell, Q. Lu, J. M. Heyes, J. Rosen, J. G. Chen, Y. Yan, F. Jiao, B. Xu, *J. Am. Chem. Soc.* **2017**, *139*, 3774-3783.
[6] J. L. White, M. F. Baruch, J. E. Pander Iii, Y. Hu, I. C. Fortmeyer, J. E. Park, T. Zhang, K. Liao, J. Gu, Y. Yan, T. W. Shaw, E. Abelev, A. B. Bocarsly, *Chem. Rev.* **2015**, *115*, 12888-12935.
[7] a) S. Neatu, J. A. Macia-Agullo, P. Concepcion, H. Garcia, *J. Am. Chem. Soc.* **2014**, *136*, 15969-15976; b) Q. Zhai, S. Xie, W. Fan, Q. Zhang, Y. Wang, W. Deng, Y. Wang, *Angew. Chem. Int. Ed.* **2013**, *52*, 5776-5779; c) S. Xie, Y. Wang, Q. Zhang, W. Deng, Y. Wang, *ACS Catal.* **2014**, *4*, 3644-3653.
[8] S. Wan, M. Ou, X. Wang, Y. Wang, Y. Zeng, J. Ding, S. Zhang, Q. Zhong, *Dalton Trans.* **2019**, DOI: 10.1039/C1039DT02507C. .
[9] a) S. Wang, Z. Ding, X. Wang, *Chem. Commun.* **2015**, *51*, 1517-1519; b) T. Takayama, H. Nakanishi, M. Matsui, A. Iwase, A. Kudo, *J. Photoch. Photobio. A* **2018**, *358*, 416-421.
[10] P. Xia, M. Antonietti, B. Zhu, T. Heil, J. Yu, S. Cao, *Adv. Funct. Mater.* **2019**, *29*, 1900093.
[11] M. Jiang, Y. Gao, Z. Wang, Z. Ding, *Appl. Catal. B: Environ.* **2016**, *198*, 180-188.
[12] S. Balegh, O. Biddulph, *Plant Physiol.* **1970**, *46*, 1-5.
[13] a) J. M. Buriak, P. V. Kamat, K. S. Schanze, *ACS Appl. Mater. Inter.* **2014**, *6*, 11815-11816; b) M. Liang, T. Borjigin, Y. Zhang, H. Liu, B. Liu, H. Guo, *ACS Appl. Mater. Inter.* **2018**, *10*, 34123-34131.
[14] a) W. Dai, H. Xu, J. Yu, X. Hu, X. Luo, X. Tu, L. Yang, *Appl. Surf. Sci.* **2015**, *356*, 173-180; b) L. Ye, D. Wu, K. H. Chu, B. Wang, H. Xie, H. Y. Yip, P. K. Wong, *Chem. Eng. J.* **2016**, *304*, 376-383.
[15] A. Li, X. Chang, Z. Huang, C. Li, Y. Wei, L. Zhang, T. Wang, J. Gong, *Angew. Chem. Int. Ed.* **2016**, *55*, 13734-13738.
